# Effects of Copper Citrate and Copper Sulfate on Intestinal Health, Muscle Fiber Traits, and Antioxidant Capacity in Weaned Pigs

**DOI:** 10.3390/ani16111615

**Published:** 2026-05-26

**Authors:** Zichen Chen, Qingtao Long, Wenjing Wang, Yiren Gu, Hui Diao, Yong Zhang, Meng Xu

**Affiliations:** 1 College of Animal & Veterinary Sciences, Southwest Minzu University, Chengdu 610041, China; 2 Key Laboratory of Qinghai-Tibetan Plateau Animal Genetic Resource Reservation and Utilization, Southwest Minzu University, Ministry of Education, Chengdu 610041, China; 3 Key Laboratory of Animal Science of National Ethnic Affairs Commission of China, Southwest Minzu University, Chengdu 610041, China; 4 Animal Breeding and Genetics Key Laboratory of Sichuan Province, Sichuan Animal Science Academy, Chengdu 610066, China; 5 College of Life Sciences and Agri-Forestry, Southwest University of Science and Technology, Mianyang 621010, China

**Keywords:** copper, tissue-specific effect, intestinal morphology, muscle fiber, antioxidant property, weaned pig

## Abstract

Copper (Cu) is an essential trace mineral additive in weaned pig diets. Copper citrate (CuCit) and copper sulfate (CuSO_4_) are common feed additives, yet their tissue-specific effects at different inclusion levels remain unclear. In this study, ninety pigs (six pens per treatment, three pigs per pen) with an initial body weight of 7.71 ± 0.15 kg were randomly assigned to five treatments for 28 d: a basal diet, and the basal diet supplemented with CuCit or CuSO_4_ at 20 or 100 mg/kg. The results show that CuCit increased slow-twitch fiber proportion in longissimus dorsi muscle (*p =* 0.009), whereas CuSO_4_ more markedly upregulated the expression of *MyHC I* (*p =* 0.005) and downregulated *MyHC IIx* (*p =* 0.006) and *MyHC IIb* (*p =* 0.033). Compared with the control group, CuCit and CuSO_4_ supplementation increased villus height and decreased crypt depth in ileum (*p <* 0.05). CuCit at 20 mg/kg showed higher *ZO-1* expression than CuSO_4_ at 100 mg/kg (*p <* 0.05). Furthermore, CuCit showed greater T-SOD (*p =* 0.018) and CAT activities (*p =* 0.002) than CuSO_4_ in muscle, as well as greater T-SOD activity (*p =* 0.011) and lower MDA content (*p =* 0.001) in ileal mucosa. These results suggest that CuCit and CuSO_4_ exhibit different tissue-specific effects in weaned pigs, providing novel insights for precision Cu supplementation.

## Simple Summary

Copper is vital for piglet health, and different supplement forms vary in effectiveness. This study aims to compare the effects of copper citrate and copper sulfate at different supplementation levels on muscle fiber traits, intestinal health, and antioxidant status in weaned pigs. Ninety pigs were randomly assigned to five treatments for 28 d: a basal diet, and the basal diet supplemented with copper citrate or copper sulfate at 20 or 100 mg/kg. At a lower dose, copper citrate strengthened the gut barrier, enhanced antioxidant defenses, and affected skeletal muscle fiber traits. In contrast, copper sulfate was needed at a higher dose to achieve similar effects. This suggests that copper citrate may achieve comparable health benefits with lower copper input, thereby helping to reduce feed costs. This work provides science-based guidance for precise, sustainable copper use in piglet nutrition.

## 1. Introduction

In modern intensive livestock production, feed cost represents one of the most significant expenses in the industry, making the enhancement of feed efficiency and the maintenance of animal health central to sustainable development [1]. Feed additives enhance profitability, welfare, and reduce environmental emissions by optimizing nutritional structure, digestion, absorption, intestinal health, and immune-antioxidant functions. Among them, trace minerals, despite low inclusion levels, are indispensable for growth, development, metabolic regulation, and overall health [2]. Copper (Cu) is an essential trace mineral that serves as a catalytic cofactor for a wide array of metalloenzymes involved in fundamental biological processes, including iron metabolism, neuropeptide synthesis, connective tissue formation, and cellular antioxidant defense systems [3]. Cu is widely used as a feed additive to enhance growth performance in animal nutrition, particularly in swine production [4]. The dosage and source of Cu affect animal performance, as an appropriate amount promotes growth and enhances production, while excessive levels can induce toxicity in animals and pose environmental risks [5]. Dietary Cu supplementation at levels exceeding nutritional requirements remains an effective strategy to enhance growth performance and feed efficiency in weaned pigs, particularly during the immediate post-weaning phase when pigs face multifaceted stress factors [6]. The efficacy of Cu supplementation is profoundly influenced by its chemical form and bioavailability. Inorganic copper sulfate (CuSO_4_) has been conventionally utilized due to its cost-effectiveness and documented benefits in modulating oxidative stress and microbiota composition, and organic Cu complexes such as copper citrate (CuCit) have garnered increasing interest due to their proposed higher absorption efficiency and metabolic stability [7,8]. Beyond these functions, Cu is essential for muscle development, intestinal integrity, and immune responses, all of which are key determinants of swine productivity [9,10].

The post-weaning period presents substantial challenges to intestinal integrity and systemic homeostasis. Weaned pigs undergo a critical transition from suckling on a sow’s milk to consuming solid feed, during which precise control of Cu supplementation in the diet is required [11]. The intestinal tract is not only the primary site for nutrient absorption but also a critical barrier against pathogens and toxins [12]. Weaning stress frequently disrupts gut architecture, impairs barrier function through dysregulation of tight junction proteins, and induces localized inflammation and oxidative stress [13]. Recent research has expanded the understanding of Cu’s biological roles beyond growth promotion. Cu contributes to intestinal health by promoting villus development, modulating immune responses, and enhancing mucosal antioxidant capacity through Cu-dependent enzymes such as SOD [9]. Concurrently, skeletal muscle development, a primary determinant of growth performance, can be influenced by trace mineral status [14]. Cu participates in muscle energy metabolism and modulates muscle fiber type composition by altering the metabolic phenotype of myofibers, thereby influencing the overall growth rate and post-fattening meat quality [15].

However, a systematic comparison of CuCit and CuSO_4_ as dietary Cu sources under practical production conditions remains limited. Previous studies have primarily focused on individual parameters such as growth performance or hematological indices, with insufficient research systematically evaluating the effects of different Cu sources on multiple physiological functions in weaned pigs within a unified experimental framework. Therefore, this study aims to systematically compare the effects of CuCit and CuSO_4_ at different supplementation levels on growth performance, muscle fiber type composition, intestinal morphology, and antioxidant status in weaned pigs. Based on the differences in absorption and metabolism between inorganic and organic Cu, we hypothesize that CuCit and CuSO_4_ differ in their effects on weaned pigs and may exert tissue-specific actions. This study provides evidence-based guidance for scientific Cu source selection and precision supplementation.

## 2. Materials and Methods

### 2.1. Animals, Design, and Diets

Animal procedures were conducted in compliance with the guiding principles of the Animal Care and Ethics Committee of Southwest Minzu University (Approval No. SMU-202307145). Ninety 28-day-old Duroc × Landrace × Yorkshire (DLY) crossbred male piglets, derived from thirty multiparous sows (parities 2 to 3), with an average body weight of 7.71 ± 0.15 kg, were divided into blocks according to initial body weight and randomly assigned to five dietary treatments, with each group comprising six replicates (pens) and three piglets per pen. The experiment began with a 3-day adaptation period, during which all pigs were fed the basal diet, which was analyzed to contain 6.8 mg/kg of Cu from intrinsic dietary sources. Following adaptation, the trial entered a 28-day formal period. Throughout the 28-day trial, the control group (CON) continued to receive the basal diet (Supplementary Materials, Table S1), and the other four groups were fed the basal diet supplemented with CuCit or CuSO_4_ at 20 or 100 mg of Cu per kg of diet. The Cu content in the diet was determined by flame atomic absorption spectrometry (Table S2). The basal diet was formulated in accordance with the recommendations of the Nutrient Requirements of Swine (2012). Feed and water were available ad libitum. All pigs were housed in an environmentally controlled room maintained at 28 ± 1 °C and 65–75% relative humidity. The CuCit (≥98.5% purity, containing ≥34.0% Cu) and CuSO_4_ (≥98.5% purity, containing ≥25.0% Cu) were provided by Sichuan Livestock Science Group Co., Ltd. (Chengdu, China).

### 2.2. Performance Measurements and Sample Collection

Pigs were weighed individually on days 0 and 28 to calculate average daily gain (ADG). Daily feed intake was recorded to determine average daily feed intake (ADFI), and feed-to-gain ratio (F/G) was derived as ADFI/ADG. On day 28, one pig per pen with body weight closest to the pen average was selected and euthanized after a 12 h feed restriction. Pigs were intramuscularly injected with Zoletil (25 mg/kg), and after confirming that they had entered a state of deep anesthesia, they were euthanized by rapid exsanguination via carotid artery incision. Tissue samples were collected immediately. The longissimus dorsi (LD) muscle was dissected from the right side between the 10th and 12th ribs, cut into cubes, and frozen in liquid nitrogen. A segment of the middle ileum was excised after ligation, flushed with ice-cold saline, and approximately 2 cm was fixed in 4% paraformaldehyde for histological assessment. Mucosa from the remaining segment was harvested with a sterile glass slide and snap-frozen in liquid nitrogen. All frozen samples were stored at −80 °C for subsequent analyses.

### 2.3. Total RNA Extraction and Real-Time Quantitative PCR

Total RNA was extracted using the RNAiso Plus reagent (TaKaRa, Dalian, China), and reverse transcription was performed according to the instructions of the PrimeScript RT reagent Kit with gDNA Eraser (TaKaRa). The integrity of the RNA was examined by agarose gel electrophoresis. Real-time quantitative PCR detection was performed with TB Green^®^ Premix Ex Taq™ II (TaKaRa) following the manufacturer’s instructions. Primer sequences were designed based on the known gene sequences available in GenBank to specifically amplify the target gene mRNA for analysis (Table S3). The amplification efficiency for the primer pair was determined from standard curves, and melting curve analysis confirmed a single product for each amplicon. Glyceraldehyde-3-phosphate dehydrogenase (GAPDH) was found to be relatively stable in this experiment and was used as an internal reference gene to normalize expression levels. Relative mRNA abundance was calculated using the 2^–ΔΔCT^ method.

### 2.4. Immunofluorescence Homology Double-Labeling Assay

Longissimus dorsi samples were fixed, dehydrated, cleared, and embedded in paraffin, then sectioned at 10 μm. Following dewaxing, rehydration, and microwave-assisted antigen retrieval in EDTA buffer (pH 8.0), sections were rinsed in PBS and treated with 3% H_2_O_2_ to block endogenous peroxidase. After blocking with 3% BSA, double immunofluorescence labeling was performed: sections were incubated overnight at 4 °C with anti-slow MyHC primary antibody, followed by a secondary antibody for 1 h at room temperature. After washing with TBST and repeating antigen retrieval to remove the first antibody complex, sections were incubated overnight at 4 °C with anti-fast MyHC primary antibody, followed by its corresponding secondary antibody. Nuclei were counterstained with DAPI. After applying autofluorescence quencher and washing, sections were mounted with anti-fade medium and examined under fluorescence microscopy. Slow-twitch fibers exhibited green fluorescence, fast-twitch fibers red fluorescence. Fields of view were systematically selected from the central region of each section to avoid edge artifacts. For each sample, three random fields of view were captured at 100× magnification. Percentage of slow-twitch fibers = (number of slow fibers/total number of fibers) × 100. All observations and counts were performed by observers who were blinded to the treatment groups.

### 2.5. Antioxidant Properties

Tissue samples from the LD muscle and ileal mucosa were processed for biochemical analysis. The LD muscle and ileal mucosa were directly homogenized. Both tissue types were then homogenized in ice-cold PBS at a 1:9 (*w*/*v*) ratio using a tissue homogenizer. The homogenates were centrifuged at 2500× *g* for 15 min at 4 °C. Protein concentration in tissue homogenate supernatants was determined using a bicinchoninic acid assay kit (Jiancheng, Nanjing, China) according to the manufacturer’s instructions. Bovine serum albumin standards were used to generate a standard curve. All antioxidant parameters, including MDA content and the activities of T-SOD, CAT, and T-AOC, were examined in accordance with the manufacturer’s protocols provided with the respective commercial assay kits (Jiancheng), and normalized to protein content. MDA content is expressed as nmol/mg protein, and enzyme activities (T-SOD, CAT, and T-AOC) are expressed as U/mg protein.

### 2.6. Histological Analysis

Ileal tissue segments were processed for histological examination. Following fixation, the samples were dehydrated, cleared, and embedded in paraffin wax. Serial sections of 10 μm thickness were prepared and stained with hematoxylin and eosin (H&E). Morphological evaluation was performed using an optical microscope (Nikon Eclipse E100, Tokyo, Japan). The measurements on villus height (V) and crypt depth (C) were done on at least 10 villus and 10 crypts that were well oriented for each sample with 100× magnification using the Image-Pro Plus software (version 6.0; Media Cybernetics, Bethesda, MD, USA). These were then averaged per pig for all further analyses. From these measurements, the ratio of villus height to crypt depth (V/C) was calculated. All histological measurements were averaged from multiple sections per animal, and all morphological measurements were performed by an observer blinded to the treatment groups.

### 2.7. Statistical Analysis

Data were analyzed as a randomized complete block design using the MIXED procedure of SAS (version 9.4; SAS Inst. Inc., Cary, NC, USA), with the pen and the individual pig designated as the experimental unit for growth performance and sample-based analyses, respectively. The statistical model evaluated the interaction effect and the main effect of Cu source within the four Cu-supplemented groups. The control group was included in the evaluation of individual treatment effects and the main effect of Cu level. A MIXED model was used to compare the five treatment groups, followed by Tukey’s test to assess differences between groups. Significant differences were considered at *p <* 0.05, and tendencies were considered at 0.05 < *p* ≤ 0.10.

## 3. Results

### 3.1. Growth Performance

The effects of dietary Cu sources and levels on the growth performance of weaned pigs are presented in Table 1. There were no significant interactions or main effects of Cu source and level on final body weight, ADG, and F/G (*p* > 0.05). ADFI tended to be greater at the 20 mg/kg Cu level than at 0 mg/kg (*p =* 0.081).

### 3.2. Percentage of Slow-Twitch Muscle Fibers

**Figure 1:**
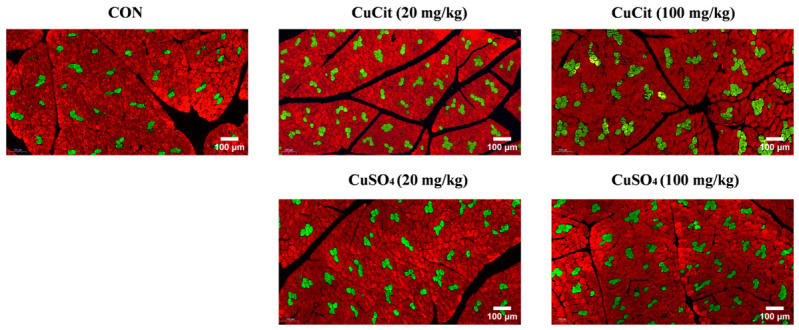
Immunofluorescence of fast (red) and slow-twitch (green) muscle fibers in LD muscle of weaned pigs (scale bar = 100 μm) (*n* = 6).

The data presented in Figure 1 and Table 2 indicate no significant interaction between Cu source and level in affecting muscle fiber type composition in the LD muscle of weaned pigs (*p =* 0.157). Weaned pigs fed CuCit had a higher percentage of slow-twitch muscle fibers than those fed CuSO_4_ (*p =* 0.009). Regarding the main effect of dietary Cu level, the 100 mg/kg treatment was higher than the 0 and 20 mg/kg treatments (*p =* 0.002).

### 3.3. The Expression of Genes Related to Muscle Fiber Types

As shown in Table 3, dietary Cu supplementation influenced the mRNA expression of genes related to muscle fiber type in LD muscle. A significant interaction between Cu source and level was detected for the expression of *MyHC I*, *MyHC IIa*, *Tnni1*, *Myoglobin*, *AMPKα1*, and *AMPKα2* (*p <* 0.05). At 20 mg/kg, the expressions of *MyHC I*, *Tnni1*, *Myoglobin*, and *AMPKα2* in CuSO_4_ groups were higher than in CuCit and CON. For *MyHC IIb* at 20 mg/kg, CuCit and CuSO_4_ maintained expression levels higher than CON, and CuCit upregulated its expression compared to CuSO_4_. At 20 mg/kg and 100 mg/kg supplementation levels, the expression of *MyHC IIx* in the CuCit group was higher than in CuSO_4_ and CON. At 100 mg/kg, CuCit and CuSO_4_ maintained the expression of *MyHC I*, *MyHC IIb*, *MyHC IIa*, and *Tnni1* levels higher than CON, while CuSO_4_ resulted in higher *MyHC IIa* expression relative to CuCit. For *AMPKα1* at 100 mg/kg, CuCit groups were higher than in CuSO_4_ and CON. Regarding the main effect of Cu source, pigs fed CuSO_4_ demonstrated upregulation of *MyHC I* (*p =* 0.005) and downregulation of *MyHC IIx* (*p =* 0.006) and *MyHC IIb* (*p =* 0.033) compared with the CuCit group. Compared with CuCit, CuSO_4_ tended to have greater *Tnni1* expression (*p =* 0.074). *PGC-1α* expression tended to be higher in the CuSO_4_ at 100 mg/kg than in the CON (*p =* 0.056). Regarding the main effect of dietary Cu level, 100 mg/kg increased the expression of *MyHC I* compared with 0 mg/kg and 20 mg/kg (*p <* 0.05), and 20 mg/kg and 100 mg/kg up-regulated *MyHC IIa*, *MyHC IIb*, *Tnni1*, and *AMPKα2* compared with 0 mg/kg (*p <* 0.05). The expression of *MyHC IIx*, *AMPKα1* and *PGC-1α* showed a tendency to be greater at 100 mg/kg than at 0 mg/kg (*p <* 0.10).

### 3.4. Muscle Antioxidant Status

As shown in Table 4, significant source and level interactions were observed for MDA content and the activities of CAT (*p <* 0.05). At 20 mg/kg, CuCit showed a lower MDA content than CON and CuSO_4_, whereas CuSO_4_ showed higher CAT activity than CON and CuCit. At 100 mg/kg, CuCit and CuSO_4_ decreased MDA content relative to the CON, with CuSO_4_ achieving the lowest values, and CuCit resulted in higher CAT activity than the CON and CuSO_4_ groups. For the main effect of source, weaned pigs fed CuCit had a higher MDA content (*p <* 0.001) and higher activities of T-SOD (*p =* 0.018) and CAT (*p =* 0.002) compared to those fed CuSO_4_. Regarding the main effect of level, pigs fed the 100 mg/kg Cu exhibited a reduction in MDA content (*p =* 0.002) and an increase in CAT activity (*p =* 0.040) compared with those fed the 0 or 20 mg/kg diets.

### 3.5. Ileal Morphology

**Figure 2:**
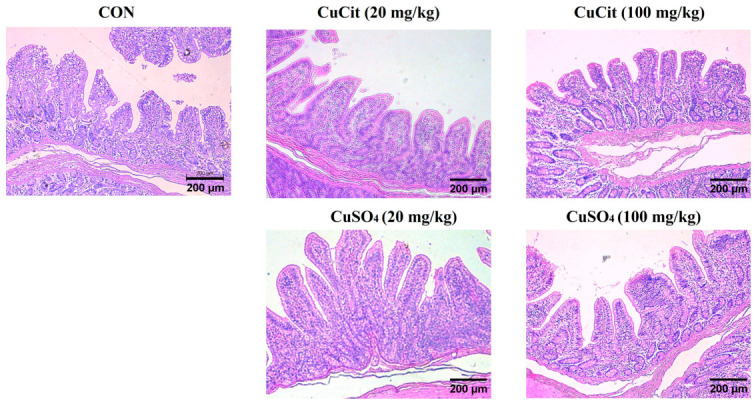
H&E staining of the ileum morphology of weaned pigs (scale bar = 200 μm) (*n* = 6).

The data presented in Figure 2 and Table 5 indicate no significant source and level interactions for villus height, crypt depth, and the ratio of villus height to crypt depth (*p* > 0.05). Dietary Cu level influenced ileal morphology. Villus height was greater in pigs fed 20 mg/kg Cu than in those fed either 0 or 100 mg/kg (*p =* 0.005). Compared with the 0 mg/kg, supplementation at 20 and 100 mg/kg resulted in a reduced crypt depth (*p =* 0.003) and an increased ratio of villus height to crypt depth (*p <* 0.001).

### 3.6. Ileal Tight Junction Gene Expression

The relative mRNA expression of tight junction genes in the ileal mucosa is presented in Table 6. Significant source and level interactions were observed for *ZO-1* and *occludin* expression (*p <* 0.05). At 20 mg/kg, CuCit upregulated *ZO-1* expression compared to the CON and CuSO_4_ groups. At 100 mg/kg, CuSO_4_ enhanced *ZO-1* expression relative to CON and CuCit groups. Notably, compared with the CuCit group at 20 mg/kg, the CuSO_4_ group at 100 mg/kg exhibited lower *ZO-1* expression.

### 3.7. Ileal Inflammatory Gene Expression

As presented in Table 7, the expression of inflammation genes in ileal mucosa was not significantly altered by dietary Cu treatments. There were no significant interactions or main effects of source and level detected for *IL-1β* and *TNF-α* expression (*p* > 0.05). Compared with CuSO_4_, CuCit tended to have greater *IL-10* expression (*p =* 0.093).

### 3.8. Ileal Antioxidant Status

As shown in Table 8, the antioxidant status in the ileal mucosa was affected by dietary Cu supplementation. Significant source and level interactions were detected for MDA content (*p <* 0.001). At 20 and 100 mg/kg supplementation levels, MDA content was reduced in the CuCit and CuSO_4_ groups compared to the CON, with the CuCit groups showing a further reduction relative to the CuSO_4_ groups. Analysis of the main effects revealed that pigs fed CuCit had lower MDA content (*p =* 0.001) and higher T-SOD activity (*p =* 0.011) in the ileal mucosa compared to those fed CuSO_4_. Regarding the main effect of level, the results revealed that 20 and 100 mg/kg supplementation reduced MDA content and enhanced T-AOC compared to the 0 mg/kg level (*p <* 0.001).

## 4. Discussion

CuCit and CuSO_4_ are widely utilized Cu supplements in livestock feed, recognized for their efficacy in enhancing growth performance and promoting overall health [6]. Dietary Cu supplementation did not significantly improve the final body weight, ADG or F/G, but ADFI tended to be greater at 20 mg/kg than at 0 mg/kg. This suggests that weaned pigs may prefer Cu-supplemented diets, consistent with previous findings [16]. The basal diet in this study already contained 6.8 mg/kg Cu from the mineral premix, which is close to the NRC (2012) requirement estimate for weaned pigs. It has been suggested that dietary Cu levels of 100–250 mg/kg in weaned pigs are typically required to produce significant improvements in growth performance [17]. In the present study, the supplemented levels of 20 and 100 mg/kg may not have attained the requisite threshold to elicit a significant growth-promoting effect.

Muscle development is a key determinant of the economic returns in swine production, with feed nutrition serving as a central approach to regulating these processes [18]. Skeletal muscle development and fiber type composition serve as a fundamental physiological basis influencing livestock growth efficiency and meat production performance [19]. The proportion of different muscle fiber types, which are defined by their specific MyHC isoforms, dictates the contractile and metabolic properties of the muscle and influences broader physiological processes during growth [20]. Slow-twitch muscle fibers are more conducive to efficient energy utilization and sustained protein synthesis, attributable to their higher myoglobin content, greater oxidative metabolic capacity, lower glycogen levels, and finer diameter [21]. Muscle fiber type composition is regulated by the AMPK signaling pathway [22]. As critical subunits mediating AMPK activation, *AMPKα1* and *AMPKα2* support the slow-twitch phenotype by enhancing oxidative metabolic genes and activating PGC-1α, which is a key regulator of mitochondrial biogenesis and slow fiber gene expression [22,23]. Studies have shown that Cu as a feed additive can promote muscle growth and development in livestock and poultry, potentially by mediating the AMPK signaling pathway and thereby promoting mitochondrial fission and *PGC-1α* expression [24]. Our results suggest that CuCit and CuSO_4_ increased the expression of *AMPKα1* and *AMPKα2* in muscle. CuCit significantly increased the proportion of slow-twitch fibers relative to CuSO_4_, whereas CuSO_4_ upregulated slow-twitch muscle gene expression compared with CuCit. And at the 20 mg/kg supplementation level, CuSO_4_ significantly increased the expression of slow-twitch-related genes, including *MyHC I*, *Tnni1*, *Myoglobin*, and *AMPKα2*, compared with CuCit. At these two Cu supplementation levels, CuCit increased the expression of *MyHC IIx* and *MyHC IIb* compared with CuSO_4_. However, the difference in the regulation of slow-twitch muscle genes between CuCit and CuSO_4_ diminished at 100 mg/kg. These differences may be due to the temporal and hierarchical regulation of muscle fibers: transcriptional changes may precede phenotypic remodeling, and changes in mRNA may precede or lag behind changes in proteins [25,26]. Cu plays an important regulatory role in skeletal muscle development [27]. In this study, Cu supplementation enhanced the expression of the fast-twitch fiber genes *MyHC IIa* and *MyHC IIb*, as well as the slow-twitch fiber genes *MyHC I*, *Tnni1*, and *AMPKα2*. Although there is currently limited research directly exploring the correlation between Cu supplementation and muscle fiber types, previous studies have confirmed that Cu is essential for myoblast proliferation and differentiation [28]. Furthermore, dietary supplementation with organic Cu has been shown to significantly affect the number of type I, IIa, and IIb muscle fibers in finishing pigs [29]. These results suggest that inorganic and organic Cu may participate in the expression of muscle fiber type genes through different regulatory mechanisms, and further investigation is needed to clarify the differences in the mechanisms.

Early weaning can lead to a reduction in villus height, an increase in crypt depth, and a compromise in intestinal barrier function [30]. Dietary Cu supplementation improves intestinal morphology in weaned pigs, notably by increasing villus height and reducing crypt depth, which supports enhanced digestion, absorption, and nutrient utilization following weaning [31]. This study found that ileal morphology was primarily influenced by Cu level rather than Cu source. Intestinal absorption of Cu is primarily mediated by transporters such as copper transporter 1 (CTR1) in intestinal epithelial cells, a process characterized by saturability and carrier-mediated kinetics [32,33]. Cu level directly regulates the proliferation rate of intestinal epithelial cells, affecting Cu absorption, whereas the differences between Cu sources may become apparent at specific doses [34,35,36]. However, when Cu levels exceed their absorption and transport capacity, it may interfere with the physiological functions of Cu by affecting intestinal barrier function and cell viability [34,35]. In the present study, villus height was greater at 20 mg/kg than at 100 mg/kg. Owing to the differential intestinal absorption efficiency between organic and inorganic Cu, the dose thresholds at which they exert beneficial effects are also distinct [34,37]. Organic Cu upregulates peptide transporter 1 and the amino acid transporter in the intestine, resulting in higher intestinal absorption efficiency compared with inorganic Cu within a certain threshold [38]. Meanwhile, CuCit at 20 mg/kg showed higher *ZO-1* expression than CuSO_4_ at 100 mg/kg. The results of this trial are consistent with those of previous similar studies, indicating that although organic and inorganic Cu can improve intestinal morphology in weaned pigs, the effective doses may differ [39,40]. The cytokine IL-10 is a key anti-inflammatory cytokine that plays an important role in suppressing excessive intestinal inflammatory responses and maintaining mucosal immune homeostasis [41]. The trend toward higher *IL-10* expression in the CuCit group suggests that CuCit may have greater potential than CuSO_4_ in enhancing the intestinal anti-inflammatory capacity of weaned pigs [42].

Cu is established as a pivotal micronutrient in antioxidant defense [43]. As a cofactor for antioxidant enzymes such as Cu/Zn-SOD, Cu at appropriate levels reduces lipid peroxidation by enhancing the activity of these enzymes [6]. The antioxidant status is typically assessed by biomarkers such as MDA for lipid peroxidation, and T-SOD, CAT, and T-AOC to evaluate the antioxidant defense system [44]. In this study, CuCit and CuSO_4_ enhanced the antioxidant capacity of the LD muscle compared to the CON group, among which the CuCit group exhibited higher T-SOD and CAT activities but relatively higher MDA content compared to the CuSO_4_ group. This observation may be attributable to differential temporal kinetics in the antioxidant responses induced by distinct Cu sources, meaning that although CuCit exhibits increased activities of T-SOD and CAT, its effect on MDA may exhibit a time delay [45]. Furthermore, the number of animal replicates used for tissue-level analysis in this study was relatively limited, and sampling was performed at a single time point, which may have restricted the observation of potential differences in MDA or enzyme activities between CuCit and CuSO_4_. Although our data indicates the two Cu sources effectively alleviated oxidative stress in muscle compared with the CON, more refined comparisons between CuCit and CuSO_4_ require further investigation. In ileal mucosa, CuCit demonstrated a distinct advantage over CuSO_4_ and showed lower MDA content and higher T-SOD activity than CuSO_4_. These results further indicate that the effects of CuCit and CuSO_4_ are tissue-specific. Organic and inorganic Cu have different solubility in the gastrointestinal tract, which may lead to differences in their absorption and utilization by intestinal cells [34]. Dietary Cu is primarily taken up by enterocytes via the high-affinity CTR1, processed intracellularly through chaperone proteins like antioxidant 1 copper chaperone, and then exported into the portal circulation by the Cu-transporting ATPase 1 [46]. Studies have found that, compared to inorganic CuSO_4_, organic Cu sources can differentially regulate these transporters and enhance *SOD1* expression [34]. In this study, CuCit significantly reduced MDA content and enhanced T-SOD activity compared with the CuSO_4_ group in ileum. These results suggest that CuCit may be associated with greater intestinal antioxidant capacity and higher intestinal utilization efficiency [8]. This interpretation is in line with previous observations in piglets and other livestock species [6,47]. Previous studies also indicate that organic Cu sources, such as Cu methionine hydroxy analog chelate, may be superior to inorganic Cu sources in terms of functional efficacy at lower supplementation levels [48].

## 5. Conclusions

Cu sources did not affect growth performance but exhibited tissue-specific effects. CuCit increased the proportion of slow-twitch muscle fibers, whereas CuSO_4_ showed stronger upregulation of slow-twitch muscle genes. Compared with CuSO_4_, CuCit exhibited higher T-SOD and CAT activities in muscle as well as higher T-SOD activity and lower MDA content in ileum. CuCit and CuSO_4_ improved intestinal morphology. And CuCit at 20 mg/kg resulted in higher *ZO-1* expression than CuSO_4_ at 100 mg/kg. These tissue-specific effects indicate that CuCit and CuSO_4_ differ in their impacts on muscle fiber traits, intestinal parameters, and antioxidant status. However, the limited sample size necessitates further studies to confirm their bioavailability and long-term effects.

## Figures and Tables

**Table 1:** Effect of dietary Cu sources and levels on growth performance of weaned pigs (*n* = 6).

Cu Source	Added Cu Level (mg/kg)	Starting Weight (kg)	Final Weight (kg)	ADG (g)	ADFI (g)	F/G
CON	0	7.71	15.34	272.60	456.80	1.68
CuCit	20	7.67	15.52	280.43	499.88	1.79
	100	7.74	15.86	290.07	503.79	1.75
CuSO_4_	20	7.70	15.44	276.29	490.62	1.79
	100	7.70	15.76	287.71	480.98	1.67
SEM		0.186	0.424	10.683	14.247	0.078
Main effect of source						
CuCit		7.70	15.69	285.25	501.84	1.77
CuSO_4_		7.70	15.60	282.00	485.80	1.73
SEM		0.171	0.414	9.206	10.243	0.063
Main effect of level						
	0	7.71	15.34	272.64	456.80 *	1.68
	20	7.69	15.48	278.36	495.00 *	1.79
	100	7.72	15.81	288.89	492.00	1.71
SEM		0.185	0.417	10.348	14.113	0.076
*p*-value for individual treatments		0.616	0.970	0.924	0.629	0.589
*p*-value for source		0.989	0.669	0.632	0.273	0.537
*p*-value for level		0.866	0.251	0.281	0.081	0.109
*p*-value for source × level		0.645	0.961	0.893	0.658	0.626

* Mean values in the same column with a significant difference tendency (0.05 < *p* ≤ 0.10).

**Table 2:** Effect of dietary Cu sources and levels on percentage of slow-twitch muscle fibers in LD muscle of weaned pigs (*n* = 6).

Cu Source	Added Cu Level (mg/kg)	Slow-Twitch Muscle Fibers (%)
CON	0	13.06
CuCit	20	16.40
	100	18.59
CuSO_4_	20	13.39
	100	16.24
SEM		0.206
Main effect of source		
CuCit		17.49
CuSO_4_		14.81
SEM		0.582
Main effect of level		
	0	13.06 ^b^
	20	14.89 ^b^
	100	17.41 ^a^
SEM		0.802
*p*-value for individual treatments		0.155
*p*-value for source		0.009
*p*-value for level		0.002
*p*-value for source × level		0.157

^a,b^ Values with different superscripts in the same column are significantly different (*p <* 0.05).

**Table 3:** Effects of dietary Cu sources and levels on the expression of genes related to muscle fiber types in LD muscle of weaned pigs (*n* = 6).

Cu Source	Added Cu Level (mg/kg)	*MyHC I*	*MyHC IIa*	*MyHC IIx*	*MyHC IIb*	*Tnni1*	*Tnni2*	*Myoglobin*	*AMPKα1*	*AMPKα2*	*PGC-1α*
CON	0	1.00 ^c^	1.00 ^d^	1.00 ^c^	1.00 ^c^	1.00 ^c^	1.00	1.00 ^b^	1.00 ^c^	1.00 ^c^	1.00 *
CuCit	20	1.42 ^c^	7.98 ^ab^	2.16 ^a^	4.33 ^a^	1.42 ^bc^	1.06	0.84 ^b^	1.28 ^bc^	1.89 ^bc^	1.43
	100	4.77 ^a^	4.55 ^c^	2.13 ^a^	4.69 ^a^	2.62 ^a^	1.41	1.32 ^ab^	2.28 ^a^	2.36 ^ab^	1.46
CuSO_4_	20	4.35 ^ab^	5.43 ^bc^	1.05 ^bc^	3.11 ^b^	2.59 ^a^	1.38	1.54 ^a^	1.87 ^ab^	3.16 ^a^	1.51
	100	4.81 ^a^	8.99 ^a^	1.71 ^c^	4.08 ^a^	2.21 ^ab^	1.13	1.08 ^ab^	1.35 ^bc^	1.82 ^bc^	2.15 *
SEM		0.343	1.120	0.188	0.339	0.171	0.096	0.114	0.136	0.188	0.225
Main effect of source											
CuCit		3.10	6.27	2.15	4.52	2.02 *	1.24	1.08	1.78	2.13	1.44
CuSO_4_		4.58	7.21	1.38	3.60	2.40 *	1.26	1.31	1.61	2.49	1.83
SEM		0.269	0.486	0.145	0.294	0.132	0.068	0.090	0.125	0.146	0.171
Main effect of level											
	0	1.01 ^b^	1.00 ^b^	1.00 *	1.00 ^b^	1.00 ^b^	1.00	1.00	1.00 *	1.00 ^b^	1.00 *
	20	2.89 ^b^	6.71 ^a^	1.60	3.72 ^a^	2.00 ^a^	1.22	1.19	1.58	2.53 ^a^	1.47
	100	4.79 ^a^	6.77 ^a^	1.92 *	4.39 ^a^	2.41 ^a^	1.27	1.20	1.82 *	2.09 ^a^	1.80 *
SEM		0.675	0.414	0.297	0.417	0.298	0.123	0.183	0.257	0.330	0.253
*p*-value for individual treatments		0.002	<0.001	0.003	<0.001	<0.001	0.044	0.011	<0.001	<0.001	0.056
*p*-value for source		0.005	0.206	0.006	0.033	0.074	0.845	0.115	0.190	0.121	0.134
*p*-value for level		0.002	0.004	0.079	<0.001	0.008	0.248	0.649	0.068	0.009	0.083
*p*-value for source × level		0.005	<0.001	0.129	0.445	0.003	0.015	0.006	0.001	0.004	0.280

^a–d^ Values with different superscripts in the same column are significantly different (*p <* 0.05). * Mean values in the same column with a significant difference tendency (0.05 < *p* ≤ 0.10).

**Table 4:** Effect of dietary Cu sources and levels on antioxidant indexes in LD muscle of weaned pigs (*n* = 6).

Cu Source	Added Cu Level (mg/kg)	MDA (nmol/mg Prot)	T-SOD (U/mg Prot)	CAT (U/mg Prot)	T-AOC (U/mg Prot)
CON	0	1.17 ^a^	9.23 ^ab^	2.28 ^d^	0.02
CuCit	20	0.37 ^d^	8.86 ^ab^	2.24 ^d^	0.02
	100	0.87 ^b^	10.46 ^a^	5.36 ^a^	0.11
CuSO_4_	20	0.61 ^c^	8.57 ^b^	3.52 ^b^	0.08
	100	0.33 ^d^	8.96 ^ab^	2.92 ^c^	0.14
SEM		0.022	0.301	0.115	0.068
Main effect of source					
CuCit		0.63	9.66	3.80	0.07
CuSO_4_		0.47	8.77	3.22	0.11
SEM		0.015	0.213	0.087	0.062
Main effect of level					
	0	1.17 ^a^	9.23	2.28 ^b^	0.02
	20	0.49 ^b^	8.72 *	2.88 ^b^	0.05
	100	0.61 ^b^	9.71 *	4.14 ^a^	0.13
SEM		0.125	0.416	0.574	0.018
*p*-value for individual treatments		<0.001	0.010	<0.001	0.469
*p*-value for source		<0.001	0.018	0.002	0.461
*p*-value for level		0.002	0.095	0.040	0.216
*p*-value for source × level		<0.001	0.077	<0.001	0.822

^a–d^ Values with different superscripts in the same column are significantly different (*p <* 0.05). * Mean values in the same column with a significant difference tendency (0.05 < *p* ≤ 0.10).

**Table 5:** Effect of dietary Cu sources and levels on the ileum morphology of weaned pigs (*n* = 6).

Cu Source	Added Cu Level (mg/kg)	Villus Height	Crypt Depth	Villus Height: Crypt Depth
CON	0	376.02	313.71	1.22
CuCit	20	473.88	226.98	2.12
	100	433.11	218.32	1.98
CuSO_4_	20	463.38	240.12	1.92
	100	369.12	191.71	1.93
SEM		23.353	20.548	0.124
Main effect of source				
CuCit		453.49	222.65	2.05
CuSO_4_		416.25	215.91	1.93
SEM		22.787	11.750	0.079
Main effect of level				
	0	376.02 ^b^	313.71 ^a^	1.22 ^b^
	20	468.63 ^a^	233.55 ^b^	2.02 ^a^
	100	401.11 ^b^	205.01 ^b^	1.95 ^a^
SEM		26.080	19.711	0.120
*p*-value for individual treatments		0.101	0.356	0.578
*p*-value for source		0.231	0.694	0.310
*p*-value for level		0.005	0.003	<0.001
*p*-value for source × level		0.578	0.191	0.571

^a,b^ Values with different superscripts in the same column are significantly different (*p <* 0.05).

**Table 6:** Effect of dietary Cu sources and levels on the expression of tight-junction genes in ileal mucosa of weaned pigs (*n* = 6).

Cu Source	Added Cu Level (mg/kg)	*ZO-1*	*Occludin*
CON	0	1.00 ^c^	1.00
CuCit	20	2.12 ^a^	1.25
	100	1.05 ^c^	0.92
CuSO_4_	20	1.04 ^c^	0.86
	100	1.82 ^b^	1.28
SEM		0.115	0.106
Main effect of source			
CuCit		1.59	1.08
CuSO_4_		1.43	1.07
SEM		0.216	0.100
Main effect of level			
	0	1.00	1.00
	20	1.58	1.06
	100	1.44	1.10
SEM		0.281	0.146
*p*-value for individual treatments		<0.001	0.003
*p*-value for source		0.621	0.915
*p*-value for level		0.283	0.916
*p*-value for source × level		<0.001	0.002

^a–c^ Values with different superscripts in the same column are significantly different (*p <* 0.05).

**Table 7:** Effect of dietary Cu sources and levels on the expression of inflammation-related genes in ileal mucosa of weaned pigs (*n* = 6).

Cu Source	Added Cu Level (mg/kg)	*IL-1β*	*IL-10*	*TNF-α*
CON	0	1.00	1.00	1.00
CuCit	20	0.85	1.52	1.06
	100	1.06	1.34	0.85
CuSO_4_	20	1.12	1.14	1.06
	100	1.07	1.21	0.95
SEM		0.180	0.130	0.093
Main effect of source				
CuCit		0.95	1.43 *	0.95
CuSO_4_		1.09	1.18 *	1.01
SEM		0.12	0.095	0.068
Main effect of level				
	0	1.00	1.00	1.00
	20	0.98	1.33	1.06
	100	1.06	1.28	0.89
SEM		0.174	0.145	0.087
*p*-value for individual treatments		0.479	0.334	0.625
*p*-value for source		0.388	0.093	0.577
*p*-value for level		0.900	0.216	0.201
*p*-value for source × level		0.461	0.301	0.732

* Mean values in the same column with a significant difference tendency (0.05 < *p* ≤ 0.10).

**Table 8:** Effect of dietary Cu sources and levels on antioxidant indexes of the ileal mucosa of weaned pigs (*n* = 6).

Cu Source	Added Cu Level (mg/kg)	MDA (nmol/mg Prot)	T-SOD (U/mg Prot)	T-AOC (U/mg Prot)
CON	0	1.03 ^a^	482.14	0.02
CuCit	20	0.32 ^d^	526.05	0.03
	100	0.33 ^d^	512.17	0.04
CuSO_4_	20	0.65 ^b^	485.25	0.03
	100	0.45 ^c^	483.46	0.04
SEM		0.012	10.535	0.002
Main effect of source				
CuCit		0.32	519.11	0.03
CuSO_4_		0.55	484.36	0.04
SEM		0.033	8.167	0.001
Main effect of level				
	0	1.03 ^a^	482.14	0.02 ^c^
	20	0.49 ^b^	505.65	0.03 ^b^
	100	0.39 ^b^	497.82	0.04 ^a^
SEM		0.074	14.006	0.002
*p*-value for individual treatments		<0.001	0.571	0.623
*p*-value for source		0.001	0.011	0.844
*p*-value for level		<0.001	0.418	<0.001
*p*-value for source × level		<0.001	0.619	0.638

^a–d^ Values with different superscripts in the same column are significantly different (*p <* 0.05).

## Data Availability

Data supporting this study are available from the corresponding author upon request.

## Supplementary Materials

The following supporting information can be downloaded at: https://www.mdpi.com/article/10.3390/ani16111615/s1, Table S1: Composition of experimental diets for weaned pigs (as-fed basis). Table S2: Analyzed Cu concentrations (mg/kg) of complete diets. Table S3: Primers used for the gene expression analysis.

## Abbreviations

The following abbreviations are used in this manuscript:

Cu	Copper
CuCit	Copper citrate
CuSO_4_	Copper sulfate
ADG	Average daily gain
ADFI	Average daily feed intake
F/G	Feed-to-gain ratio
LD muscle	Longissimus dorsi muscle
MyHC	Myosin heavy chain
ZO-1	Zonula occludens-1
IL-1β	Interleukin-1 beta
IL-10	Interleukin-10
TNF-α	Tumor necrosis factor-alpha
GAPDH	Glyceraldehyde-3-phosphate dehydrogenase
MDA	Malondialdehyde
T-SOD	Total superoxide dismutase
CAT	Catalase
T-AOC	Total antioxidant capacity
H&E	Hematoxylin and eosin
SEM	Standard error of the mean
