# Supplementary material for: Effects of Copper Citrate and Copper Sulfate on Intestinal Health, Muscle Fiber Traits, and Antioxidant Capacity in Weaned Pigs
Source: Animals (Basel). 2026 May 26;16(11):1615. doi: 10.3390/ani16111615 (PMC13255900; doi:10.3390/ani16111615)
Supplement: Supplementary file 1 [file animals-16-01615-s001.zip › animals-4274357-supplementary.pdf]

Supplementary Table S1. Composition of experimental diets for weaned pigs (as-fed basis).

Item	Amount
Ingredient (%)	
Corn	38.00
Cooked rice	28.00
Wheat bran	2.00
Extruded soybean meal ((35.8%)	5.00
Fish meal (65%)	3.00
Soybeanmeal (46%)	6.00
Soy protein concentrate	4.00
Yolk powder	3.00
Flour	2.00
Salt	0.25
Choline chloride (50%)	0.10
Dicalcium phosphate	0.80
Limestone	0.50
Glucose	2.50
Sucrose	2.50
L-Lysine-HCl	0.69
DL-Methionine	0.35
L-Threonine	0.35
L-Tryptophan	0.11
L-Valine	0.35
Nursery pig premix[1]	0.50
Total	100.00
Calculated energy and nutrient contents[2]	
Dry matter	88.46
Metabolizable energy (Mcal/kg)	3.41
Crude protein (%)	17.07
Crude fat (%)	3.70
Calcium (%)	0.82
Phosphorus (%)	0.48
Digestible Lysine (%)	1.30
Digestible Methionine (%)	0.59
Digestible Methionine+Cysteine (%)	0.80
Digestible Threonine (%)	0.85
Digestible Tryptophan (%)	0.27

[1] Provides per kilogram of diet: vitamin A 5512 IU, vitamin D_3 2250 IU, vitamin E 24 IU, vitamin K_3 3 mg, vitamin B_1 2 mg, vitamin B_2 6 mg, vitamin B_6 3 mg, vitamin B_{12} 24 μg, *D*-pantothenic acid 15 mg, nicotinic acid 14 mg, folic acid 1.2 mg, biotin 150 μg, Fe 100 mg, Zn 80 mg, Mn 25 mg, I 0.14 mg, Se 0.3 mg.

[2] Calculated energy and nutrient contents were determined based on the raw material nutrient composition data from the *TABLES OF FEED COMPOSITION AND NUTRITIVE VALUES IN CHINA (2022 THIRTY-THIRD EDITION)*.

Supplementary Table S2. Analyzed Cu concentrations (mg/kg)[1] of complete diets.

Dietary Cu source	Added Cu level (mg/kg)		
	0	20	100
CuCit	6.80	27.46	105.37
$CuSO_4$	6.80	28.02	107.83

[1] Cu element content.

Supplementary Table S3. Primers used for the gene expression analysis

Gene	Primer	Sequence（5′→3′)	GenBank ID	Product size（bp)
MyHC I	Forward	CGTGGACTACAACATCATAGGC	NM_213855.2	146
	Reverse	CCTTCTCAACAGGTGTGTCG		
MyHC IIa	Forward	CATTGAGGCCCAGAATAGGC	XM_047757183.1	127
	Reverse	TGCTTCCGTCTTCACTGTCAC		
MyHC IIx	Forward	TTGACTGGGCTGCCATCAAT	NM_001104951.2	111
	Reverse	GCCTCAATGCGCTCCTTTTC		
MyHC IIb	Forward	GACTCTGGCTTTCCTCTTTGC	XM_021066036.1	101
	Reverse	GAGCTGACACGGTCTGGAAA		
Tnni1	Forward	TGAAGCCAAATGCCTCCACAACAC	NM_213912.3	155
	Reverse	ACACCTTGTGCTTAGAGCCCAGTA		
Tnni2	Forward	GGAGAAGCAGAACTACCTGTCT	NM_001032359.1	143
	Reverse	GGACCTTGATCTCCATGTCGTA		
myoglobin	Forward	ACAGCAGAACGTCCCTTAGC	NM_214236.1	129
	Reverse	TTAAAACCGGGTGCGTCATC		
AMPKα1	Forward	CGGCAAAGTGAAGGTTGG	NM_001167633.1	123
	Reverse	AGGTTCTGAATTTCTCTGCGG		
AMPKα2	Forward	TGAGGTCGATATCTGGAGCTG	NM_214266.2	151
	Reverse	AGTGGCAACAGAACGATTGAG		
PGC-1α	Forward	CCCGAAACAGTAGCAGAGACAAG	NM_213963.2	111
	Reverse	CTGGGGTCAGAGGAAGAGATAAAG		
Nrf2	Forward	GAAAGCCCAGTCTTCATTGC	XM_021075133.1	190
	Reverse	TTGGAACCGTGCTAGTCTCA		
CAT	Forward	ACGCCTGTGTGAGAACATTG	XM_021081498.1	124
	Reverse	GTCCAGAAGAGCCTGAATGC		
ZO-1	Forward	CGTGTCAACGCCACTATCA	XM_021098848.1	90
	Reverse	TTGTCTTCCAAAGCCCCT		
Occludin	Forward	CAGTGGTAACTTGGAGGCGT	NM_001163647.2	104
	Reverse	CCGTCGTGTAGTCTGTCTC		
IL-10	Forward	TGCTCTATTGCCTGATCTTCCTG	NM_214041.1	157
	Reverse	CCCATCTGGTCCTTCGTTTG		
TNF-α	Forward	ACCTCCTCTCTGCCATCAAG	NM_214022.1	173
	Reverse	CTGCCCAGATTCAGCAAAGT		
IL-1β	Forward	ACCTGGACCTTGGTTCTC	NM_214055.1	124
	Reverse	GGATTCTTCATCGGCTTC		
GAPDH	Forward	ACTCACTCTTCTACCTTTGATGCT	NM_001206359.1	100
	Reverse	TGTTGCTGTAGCCAAATTCA		
